# In-person psychoeducational intervention to reduce rehospitalizations and improve the clinical course of major depressive disorder: a non-randomized pilot study

**DOI:** 10.3389/fpsyt.2024.1429913

**Published:** 2024-07-09

**Authors:** Dagmar Breznoscakova, Maria Pallayova, Lubomira Izakova, Maria Kralova

**Affiliations:** ^1^ Center for Mental Functions, Vranov nad Toplou, Slovakia; ^2^ Department of Social and Behavioural Medicine, Faculty of Medicine, Pavol Jozef Safarik University, Kosice, Slovakia; ^3^ 1^st^ Department of Psychiatry, University Hospital of Louis Pasteur, Kosice, Slovakia; ^4^ Department of Human Physiology, Faculty of Medicine, Pavol Jozef Safarik University, Kosice, Slovakia; ^5^ Department of Psychiatry, Faculty of Medicine Comenius University and University Hospital Bratislava, Bratislava, Slovakia

**Keywords:** depression, psychoeducation, antidepressant treatment, sickness absence, rehospitalizations

## Abstract

**Background:**

Emerging issues in the management of major depressive disorder (MDD) comprise a nonadherence to treatment and treatment failures, depressive recurrence and relapses, misidentification of incoming exacerbated phases and consequently, a chronification of depression. While antidepressant drugs constitute the standard of care for MDD, effective psychosocial interventions are needed to reduce rehospitalizations and other adverse events. The present study primarily investigated the effects and impact of implementing a structured psychoeducational intervention on the clinical course of MDD.

**Methods:**

A non-randomized comparative, pragmatic, pilot, single-center study of adults with nonpsychotic moderate or severe episode of MDD recently discharged from a psychiatric hospitalization. The consecutive subjects were allocated either to the intervention group (*N*=49) or to the attention control group (*N*=47), based on their preference. The psychoeducational intervention was based on a modified Munoz’s Depression Prevention Course. Subjects were followed up prospectively for two years.

**Results:**

The absolute changes in Beck anxiety inventory scale, Zung’s depression questionnaire, and Montgomery and Äsberg depression rating scale (MADRS) total scores at 6-month follow-up were comparable between the two groups. There were lower rates of the rehospitalization within one year (2.1% vs. 16.7%; *P*<0.001) and less rehospitalizations after one year (6.3% vs. 25%; *P*<0.001), lower rates of the ongoing sickness absence (11.5% vs. 29.2%; *P*<0.001), less persons with disability due to MDD at 1-year follow-up (1% vs. 11.5%; *P*=0.002), and less nonadherent subjects who self-discontinued treatment (6.3% vs. 28.1%; *P*<0.001) among participants in the intervention group compared to the control group. The disability due to MDD at 1-year follow-up was predicted by the absence of the psychoeducational intervention (*P*=0.002) and by the MADRS total score at 6-month follow-up (OR 1.10; 95% CI 1.003–1.195; *P*=0.044). Qualitative data indicated the intervention was desired and appreciated by the participants, as well as being practical to implement in Slovakian clinical settings.

**Conclusion:**

The results suggest the psychoeducational intervention based on a modified Munoz’s Depression Prevention Course has beneficial effects in adults with MDD recently discharged from a psychiatric hospitalization. The findings implicate the psychoeducational intervention may offer a new approach to the prevention of depressive relapses.

## Introduction

Depression is one of the most common public health challenges. The associated considerable disability and reduced quality of life underline the importance of acting on depression. The treatment of depression is multifaceted and usually involves a combination of pharmacological and non-pharmacological interventions. Nowadays, antidepressant treatment is effective, safe, and available (1). Yet, it is time-consuming, financially challenging, and human demanding. Emerging issues in the management of patients with major depressive disorder (MDD) comprise a nonadherence to treatment and treatment failures, depressive recurrence and relapses (2), misidentification of incoming exacerbated phases and consequently, a chronification of depression. As a result, we are facing a difficult question how to effectively prevent relapses of depression, rehospitalizations, and disability-related sickness absence.

Different factors appear to be associated with failure to achieve remission in patients with MDD and with subsequent relapse in patients who do achieve remission. Some of the known risk factors for depression relapse include male sex, being married or living as a couple, age of onset and severity of depressive symptomatology, number and duration of previous depressive episodes, time in remission, anxiety disorders, sexual dysfunction, patient-reported cognitive dysfunction, neuroticism, obesity, cortisol levels, childhood maltreatment, and comorbid psychiatric disorders (3–9). Despite therapeutic advances 50–70% of patients with MDD fail to achieve remission after 6–12 weeks of treatment with currently available antidepressants (9–11). According to a large STAR*D study (12) approximately a third of patients failed to achieve remission after trials of as many as four different antidepressants. In a naturalistic multisite 3-year follow-up study of initially hospitalized tertiary care patients (*N*=784) with MDD, 36% maintained remission from discharge to 3-years, and 12% of all patients never reached remission (13). Remission seems to be more likely in women, in those without a prior history of suicide attempt, and in those with lower baseline anxiety (11).

Medication non-adherence and early self-discontinuation of antidepressants are frequently reported in MDD. Data from a recent meta-analysis (14) suggest that 42% (95% confidence interval/CI 30%-54%) of individuals with MDD do not take their medication as prescribed. Nonacceptance of antidepressant treatment has been reported to be more common among patients with a low level of education (odds ratio/OR 2.6; 95% CI 1.1–5.9) and in patients who reported nonspecific possible drug side effects like fatigue, stress, and restlessness (OR 2.7; 95% CI 1.4–5.5) (3). According to a large nationally representative English primary care cohort study, early discontinuation of antidepressants increases in the post-retirement years and is higher in those without dementia and those living in urban areas (15). The findings implicate that a more active patient follow-up should be considered in these circumstances to help achieve or maintain depression remission. Alternative treatment strategies such as non-drug therapies and psychoeducational interventions may help prevent worsening of depressive symptoms both prior to and following a patient’s recovery.

According to Solmi and colleagues (16) multi-disciplinary interventions targeting both patient and prescriber, aimed at improving antidepressant adherence, include psychoeducation, psychotherapy and providing the patient with clear behavioral interventions to prevent/minimize poor adherence (16). In-person psychoeducation as a psychosocial treatment adjunct to pharmacological therapy is increasingly recognized for its value in facilitating adaption to a chronic disease diagnosis (17). Findings indicate that increased knowledge about depression and its treatment is associated with better prognosis in depression, as well as with the reduction of the psychosocial burden for the family (18). Limited evidence exists to suggest psychoeducation is effective in improving the readiness to attend treatment and medication adherence in adults with MDD.

While antidepressant drugs constitute the standard of care for MDD, effective psychosocial interventions are needed to reduce rehospitalizations and other adverse events. The present study primarily investigated the effects and impact of implementing an in-person structured psychoeducational intervention on the clinical course of nonpsychotic MDD in adults recently discharged from a psychiatric hospitalization.

## Methods

### Study design and setting

This was a non-randomized comparative, pragmatic, pilot, single-center study. The recruitment occurred during the in-hospital period. The recruited consecutive adult subjects with a nonpsychotic moderate or severe episode of MDD were allocated either to the intervention group with a psychoeducational intervention or to the attention control group, based on their preference. The intervention was based on a modified Munoz’s Depression Prevention Course (19) intended as a preventative educational experience for patients with depression. Study participants were followed up prospectively for two years.

The research was conducted in real-life settings. The authors assert that all procedures contributing to this work comply with the ethical standards of the relevant national and institutional committees on human experimentation and with the Helsinki Declaration of 1975, as revised in 2008. All procedures involving human subjects were approved by the independent Ethics committee of the Presov self-governing region (approval number: 04028/2023/0Z-45). Written informed consent was obtained from all subjects.

The report of this non-randomized study adheres to the Transparent Reporting of Evaluations with Nonrandomized Designs (TREND) recommendations (20).

### Study population

Participants were recruited from the inpatient psychiatric clinic in Slovakia. Inclusion criteria were as follows: (i) adults (≥18 years) of any sex and gender; (ii) diagnosis of a MDD with a single or repeated episodes of moderate or severe depression without psychotic features; (iii) subjects agreeing to participate to study; and (iv) provision of subject informed consent. Exclusion criteria were psychotic symptoms, higher risk of suicide (≥4 points in variable „Suicidal thoughts” of Montgomery–Åsberg depression rating scale), psychiatric comorbidity, comorbid endocrine, cerebrovascular, systemic autoimmune and other somatic diseases hampering the comprehension and following of the study, and subjects not understanding Slovak.

The diagnosis of a MDD was established by two independent psychiatrists according to International Statistical Classification of Diseases and Related Health Problems 10th Revision (ICD-10) criteria (21).

The enrolled study participants were allocated either to the intervention group with a psychoeducational intervention based on a modified Munoz’s Depression Prevention Course or to the attention control group. The group allocation was based on the preference of each subject. The participants in the intervention group actively participated in psychoeducational sessions. Only subjects who completed at least three of four sessions of the psychoeducational intervention were included in the final analysis. The attention control group did not receive the psychoeducational intervention. Subjects in both groups were receiving antidepressant medications (selective serotonin reuptake inhibitors, serotonin and noradrenaline reuptake inhibitors, other antidepressants) in line with the standard guidelines.

Recruitment of participants, the psychoeducational intervention and evaluations were performed by psychiatrists, psychotherapists, and psychologists.

### Study visits

Data on anxiety, depression, and clinical course were collected at study visits.

The baseline visit (V1) was conducted towards the end of the initial hospitalization. Psychopathological symptoms of participants were evaluated in all recruited subjects based on clinical interviews and questionnaires. The subjects in the intervention group attended the first session of the psychoeducational intervention.

The first follow-up visit (V2) occurred six months after hospital discharge, following the completion of the intervention. The second follow-up visit (V3) occurred 12 months (one year) following the hospital discharge/intervention. Questionnaire-based changes in psychopathology were evaluated in both groups at the 6-month follow up. Clinical, treatment, and outcome data (including data on psychiatric rehospitalization/unplanned hospital readmission and sickness absence) were collected from electronic medical records.

The final follow-up visit (V4) was an electronic visit based on available medical records 24 months following the hospital discharge/intervention.

#### Psychoeducational intervention

The psychoeducational intervention in the present study was based on a modified Munoz’s Depression Prevention Course (19) intended as a preventative educational experience for patients with depression. The Slovak version/the Slovak translation of the original Munoz’s Depression Prevention Course was published by Kühner Christine and Weber Iris in 2003 (22). A Slovak manual for the therapist and a manual for the patient were created and are part of the Kühner’s publication. While the original course consisted of eight sessions, the present intervention was condensed into four sessions. The Slovak manuals for the modified Munoz’s Depression Prevention Course were created and are not published. Each intervention session was intended to be delivered once a week. One session lasted approximately 90 minutes. It consisted of the theoretical part (45 minutes) and the practical part (45 minutes) with a short break in between. The intervention was delivered to small groups of 5–8 subjects. The topics of the sessions were as follows:

Session 1: Initial education, introduction to the theoretical base of depression, covering some of the basics of how to prevent depression, exercises from book learning to real-life.

Session 2: How thoughts influence mood, learning to change your thoughts.

Session 3: How activities affect mood, increasing pleasant activities.

Session 4: How contacts with people affect mood, increasing interpersonal activities, planning for the future: preventing depression.

Each session consisted of readings, discussion, review, in-class exercise, feedback from participants, homework assignment and encouraging study subjects to practice relaxation and other exercises at home on regular basis, begin self-monitoring, tracking and keeping track of mood and thoughts. The main goal was to gain greater control over one’s mood by teaching individuals to use cognitive approaches based on social learning theory, social skills training and increasing pleasant activities. The intervention was to be completely delivered over a 1-month period. The psychoeducational intervention sessions were provided by psychiatrists, psychotherapists, and psychologists. All of the staff has been trained to perform the intervention and carry out a range of associated tasks. To increase compliance and adherence, subjects were phone called and reminded about the follow-up visits.

#### Evaluation of anxiety and depression

The Beck Anxiety Inventory (BAI) (23, 24) was used as a self-report measure of anxiety. The BAI is a 21-item self-report instrument for assessing the severity of anxiety in adults with psychiatric disorders. The BAI is scored by summing the severity ratings across all 21 symptoms. The total scores can range from 0 to 63. A score of 0–7 indicates normal state, 8–15 indicates mild anxiety, 16–25 indicates moderate anxiety and 30–63 indicates severe anxiety (23).

The Zung Self-Rating Depression Scale (SDS) was administered to assess affective, cognitive, behavioral, and somatic symptoms of depression (25, 26). The SDS is a 20-item measure, with each item rated on a 4-point scale. The summary score of <50 is considered normal. Ranges for mild to moderate depression, moderate to severe depression, and severe to extreme depression are 50–59, 60–69, and 70 and over, respectively (26). An index for the SDS (derived from the summary score divided by a maximum possible score of 80) ranges from 0.25 to 1.

The Montgomery–Åsberg Depression Rating Scale (MADRS) (27, 28), a 10-item diagnostic questionnaire, was used to objectively measure the severity of depressive episodes in study participants. The rating was based on a clinical interview moving from broadly phrased questions about 10 symptoms (apparent sadness, reported sadness, inner tension, reduced sleep, reduced appetite, concentration difficulties, lassitude, inability to feel, pessimistic thoughts, suicidal thoughts) to more detailed ones for a more precise rating of severity (27). Each MADRS item yields a score of 0–6, and the overall score ranges from 0 to 60. The summary score of <7 is considered normal (depressive symptom absent). Ranges for mild depression, moderate depression, and severe depression are 7–19, 20–34, and 35–60, respectively (27).

#### Outcome measures

The primary outcome measures included baseline and 6-month follow up summary scores of the three diagnostic questionnaires (BAI, SDS, MADRS) for evaluation of the anxiety and depression (see herein). For all questionnaires, summary scores were used for statistical analysis. The secondary outcomes were the antidepressants self-discontinuation at 1-year follow-up, the rates of the rehospitalization within one year (at 1-year follow-up) and of the rehospitalization after one year (at 24-month follow-up) following the hospital discharge/intervention, the ongoing sick leave since the baseline psychiatric hospitalization discharge, and the disability due to MDD at 1-year follow-up.

### Statistical analyses

The descriptive and interferential statistics was used for the data analysis. Categorical data are presented as absolute and relative counts. Interval data are presented as mean ± standard deviation or median and interquartile range. Comparison of interval variables between two groups has been performed by Student t-test or Mann-Whitney U-test and categorical variables by chi-squared test. Changes in the values of the patient outcomes in each group were compared over time using paired t-tests (for normally distributed data) or Wilcoxon matched-pairs signed-rank test (for non-normally distributed data) for dependent samples. The relationship between outcome variables and their predictors was explored by logistic regression for categorical dependent variables. The postestimation marginal means, predictive margins, and marginal effects were computed. The prediction graphs were created to reflect the adjusted predicted probabilities of the dependent variables of interest along with their 95% CIs for the significant outcomes. Besides the stratification, multiple regression analyses were performed to determine whether unadjusted associations persisted after controlling for potential confounders. Findings were considered to be statistically significant at the 5% level. Statistical analyses were performed using Stata Special Edition statistical software Version 13.1 (StataCorp LP, College Station, TX).

## Results

**Figure 1:**
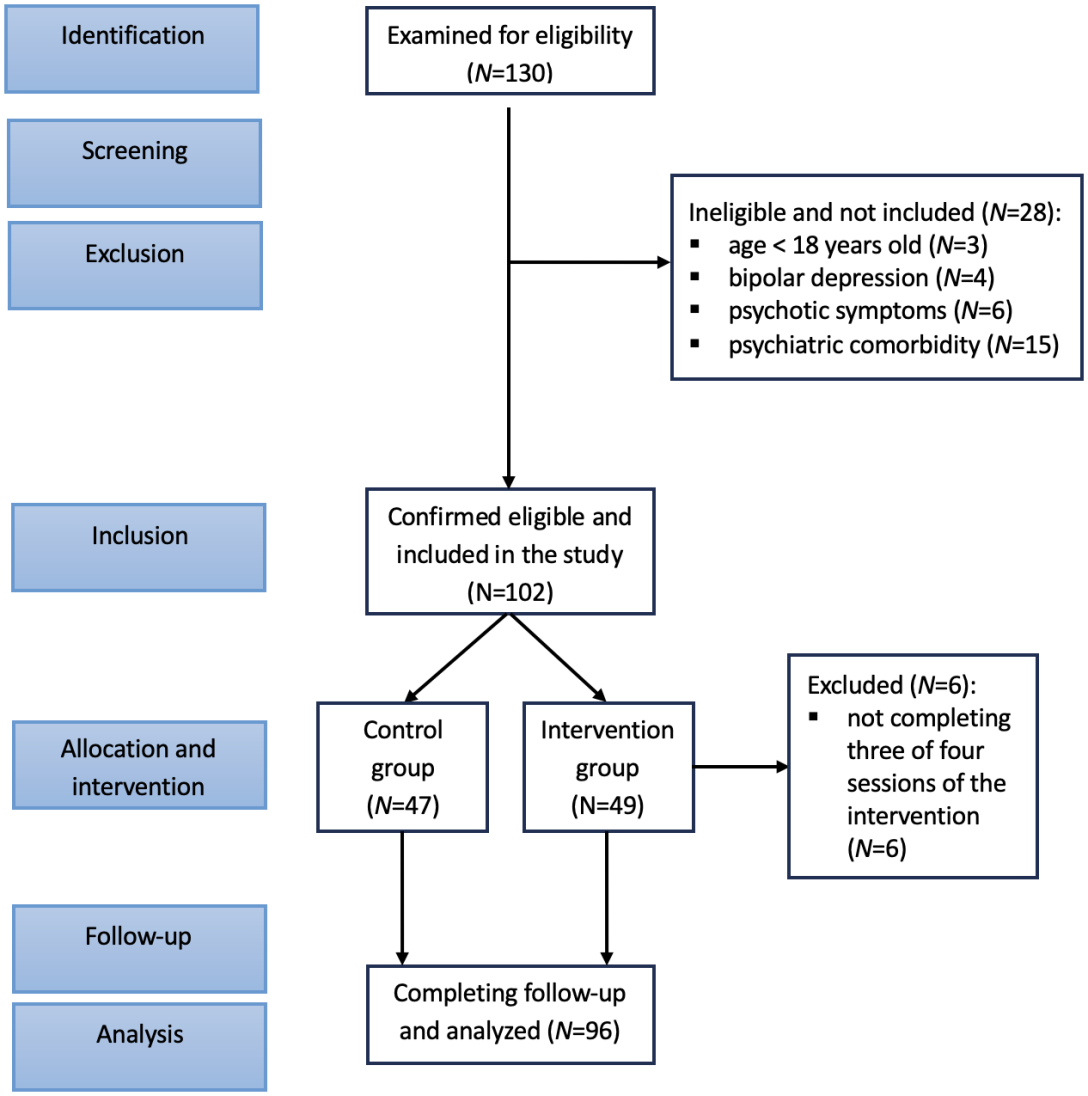
STROBE flow diagram.

**Figure 2:**
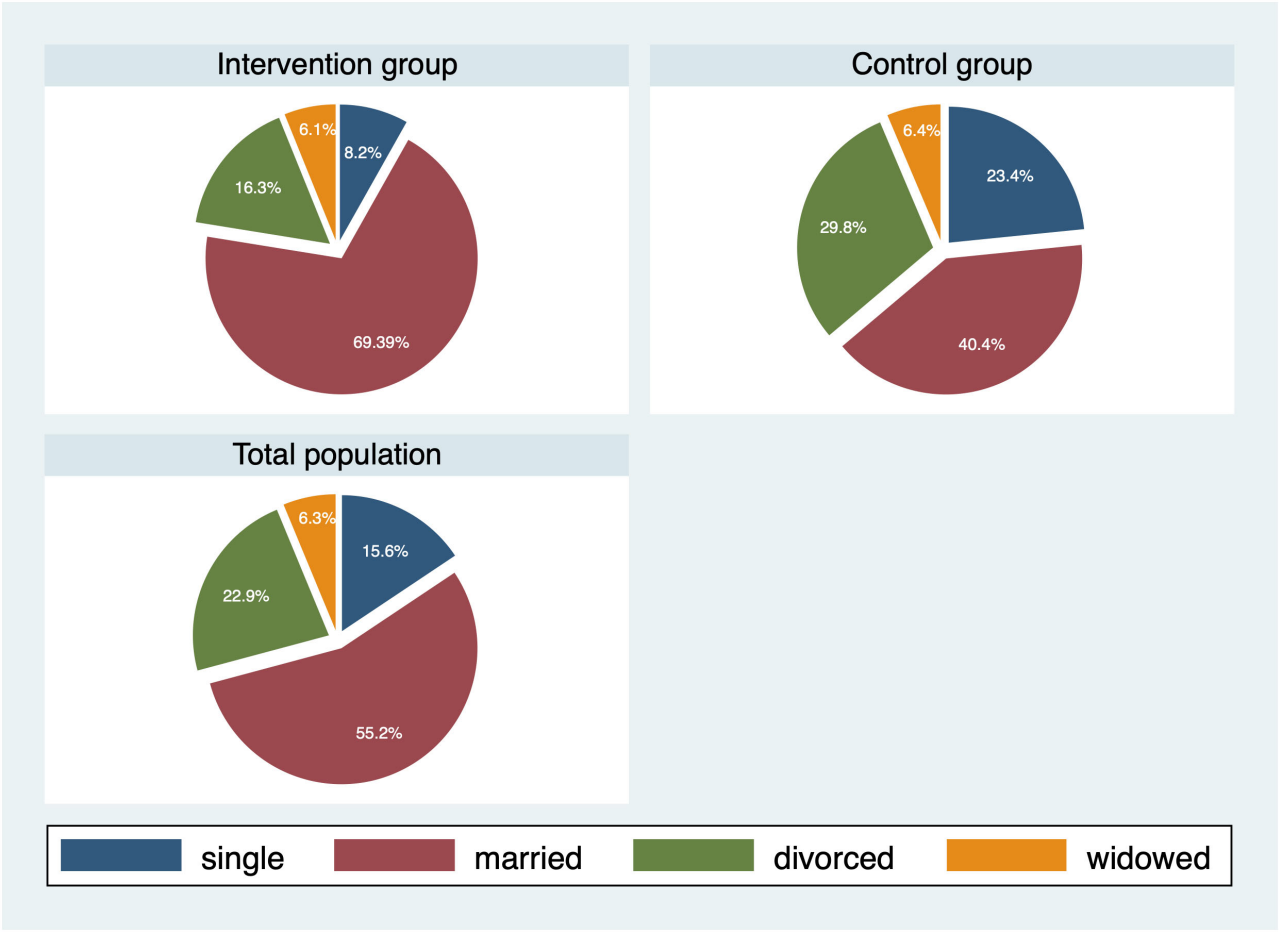
Marital status of the study participants.

**Figure 1** depicts the participant flow through each stage of the study. The study comprised 102 subjects who were initially recruited. The intervention group consisted of 49 participants, and the attention control group included 47 individuals. Six subjects were excluded from final analyses for they did not complete at least three out of four sessions of the psychoeducational intervention. **Table 1** presents the baseline demographic and clinical characteristics of study participants with comparisons between the two study groups. The cohort was predominantly female (74%) and middle-aged, with an age range of 21–74 years. There was no statistically significant difference in age and in body mass index (BMI) between the intervention group and the control group. Out of 96 study participants, 34 (35.4%) were diagnosed with recurrent depressive disorder with moderate or severe current episode (without manifested psychotic symptoms), and 62 (64.6%) subjects with nonpsychotic moderate or severe single depressive episode. Both the intervention and the control group were similar with respect to severity of depressive disorder based on 2024 ICD-10-CM Codes (**Table 1**) (29). Regarding the marital status (**Figure 2**), more subjects in the intervention group were married//in a domestic partnership and less were single or divorced compared to the control group (*P*=0.028).

**Table 1:** Baseline demographic and clinical characteristics of study participants.

	Total population (*N*=96)	Control group *(N*=47)	Intervention group *(N*=49)	*P*-value
Age, years	49 ± 12.2	49 ± 12.0	48 ± 12.5	0.934
BMI, kg/m^2^	25.8 ± 5.19	26.3 ± 5.27	25 ± 5.05	0.274
Sex				
• Males	25 (26)	13 (13.5)	12 (12.5)	0.724
• Females	71 (74)	34 (35.4)	37 (38.5)	
Diagnosis*				
• F32.1	47 (49)	22 (22.9)	25 (26)	0.451
• F32.2	15 (15.6)	6 (6.3)	9 (9.4)	
• F33.1	26 (27.1)	16 (16.7)	10 (10.4)	
• F33.2	8 (8.3)	3 (3.1)	5 (5.2)	
Level of education				
• Primary education	12 (12.5)	5 (5.2)	7 (7.3)	0.150
• Secondary education	72 (75)	39 (40.6)	33 (34.4)	
• Tertiary education	12 (12.5)	3 (3.1)	9 (9.4)	
Marital status				
• single	15 (15.6)	11 (11.5)	4 (4.2)	0.028
• married/in a domestic partnership	53 (55.2)	19 (19.8)	34 (35.4)	
• divorced	22 (22.9)	14 (14.6)	8 (8.3)	
• widowed	6 (6.3)	3 (3.1)	3 (3.1)	

*2024 ICD-10-CM Codes (29).

Data expressed as N (%) or mean ± standard deviation.

P-values are based on independent t-test or Mann-Whitney U-test for continuous and chi–square test for categorical variables.

BAI, Beck Anxiety Inventory; BMI, body mass index; MADRS, Montgomery-Äsberg Depression Scale; N, number; SDS, Zung Self-Rating Depression Scale.

**Table 2** shows antidepressant medications used at baseline and at 6-month follow-up. The psychopharmacological treatment did not change at 6-month follow-up. The 1-year follow-up medications were not examined.

**Table 2:** Antidepressant treatment at baseline and at 6-month follow-up.

	Total population (*N*=96)	Control group *(N*=47)	Intervention group *(N*=49)
SSRI	45 (46.9)	20 (20.8)	25 (26.1)
SNRI	31 (32.3)	21 (21.9)	10 (10.4)
NaSSA	2 (2.1)	0 (0)	2 (2.1)
NaRI	5 (5.2)	1 (1)	4 (4.2)
SARI	12 (12.5)	3 (3.1)	9 (9.4)
NDRI	3 (3.1)	1 (1)	2 (2.1)
TCA	5 (5.2)	3 (3.1)	2 (2.1)
combination antidepressants	9 (9.4)	3 (3.1)	6 (6.3)

Data expressed as N (%).

SSRI, Selective Serotonin Reuptake Inhibitors.

SNRI, Serotonin and Noradrenaline Reuptake Inhibitors.

NaSSA, Noradrenergic and Specific Serotonergic Antidepressants.

NaRI, Selective Noradrenaline Reuptake Inhibitors.

SARI, Serotonin Antagonist and Reuptake Inhibitors.

NDRI, Noradrenaline and Dopamine Reuptake Inhibitors.

TCA, Tricyclic Antidepressants.

**Table 3** presents the questionnaires’ summary scores at baseline and at 6-month follow-up in the two groups. Despite the comparable severity of MDD, compared to the intervention group the subjects in the control group had higher baseline total scores of the BAI (*P*=0.043), SDS (*P*=0.019), and MADRS (*P*<0.001). Compared to the control group, the intervention group had lower 6-month follow-up scores of BAI (*P*=0.043), SDS (*P*=0.011), and MADRS (*P*<0.001) questionnaires. We observed significant and clinically meaningful follow-up improvements in all patient-reported outcome measures (decreases in the BAI, SDS, and MADRS total scores) in both groups. Compared to females, males in the intervention group had greater improvements/decreases in the MADRS total score at 6-month follow-up (*P*=0.044). Importantly, there were lower rates of the rehospitalizations within one year (at 1-year follow-up) (*P*<0.001) and after one year (at 24-month follow-up) (*P*<0.001), lower rates of the ongoing sickness absence since the baseline psychiatric hospitalization discharge (*P*<0.001), less persons with disability due to MDD at 1-year follow-up (*P*=0.002), and less subjects who self-discontinued treatment (*P*<0.001) in the intervention group (**Table 4**).

**Table 3:** Questionnaires’ total scores at baseline and at 6-month follow-up in the two groups.

	Baseline	6-months follow-up	*P*-value
Control group (N=47)			
BAI score	23 (15–36)	16 (11–22)	<0.001
SDS score	70 (65–73)	51 (45–59)	<0.001
MADRS total score	40 (34–45)	18 (14–26)	<0.001
Intervention group (N=49)			
BAI score	21 (13–30)	12 (8–21)	<0.001
SDS score	63 (56–68)	47 (41–54)	<0.001
MADRS total score	32 (28–40)	13 (9–18)	<0.001

Data expressed as median (interquartile range).

P-values are based on Wilcoxon matched-pairs signed-rank test.

BAI, Beck Anxiety Inventory; MADRS, Montgomery-Äsberg Depression Scale; N, number; SDS, Zung Self-Rating Depression Scale.

**Table 4:** Comparisons of the follow-up outcomes between the two groups.

	Total population (*N*=96)	Control group (*N*=47)	Intervention group (*N*=49)	*P*-value
Ongoing sick leave at 6-month follow-up				
Yes	39 (40.62)	28 (29.17)	11 (11.46)	<0.001
No	57 (59.38)	19 (19.79)	38 (39.58)	
Antidepressants self-discontinuation at 1-year follow-up				
Yes	33 (34.38)	27 (28.12)	6 (6.25)	<0.001
No	63 (65.62)	20 (20.83)	43 (44.79)	
Rehospitalization within one year (at 1-year follow-up)				
Yes	18 (18.75)	16 (16.67)	2 (2.08)	<0.001
No	78 (81.25)	31 (32.29)	47 (48.96)	
Rehospitalization after one year (at 24-month follow-up)				
Yes	30 (31.25)	24 (25.0)	6 (6.25)	<0.001
No	66 (68.75)	23 (23.96)	43 (44.79)	
Disability due to MDD at 1-year follow-up				
Yes	12 (12.5)	11 (11.46)	1 (1.04)	0.002
No	84 (87.5)	36 (37.5)	48 (50)	

Data expressed as N (%).

P-values are based on the chi-squared test.

MDD, major depressive disorder; N, number.

**Figure 3:**
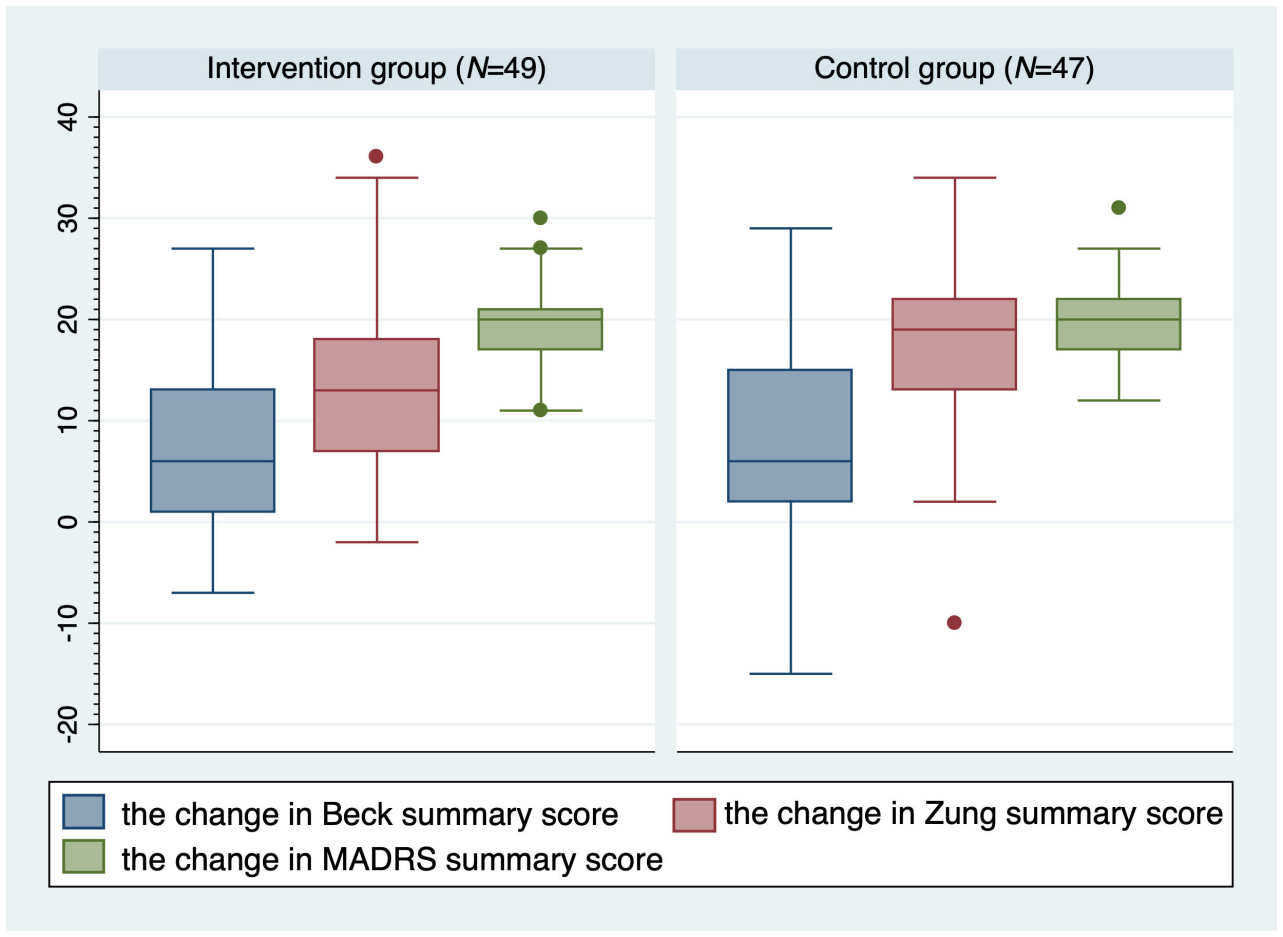
Absolute changes in Beck, Zung, and MADRS total scores at 6-month follow-up with comparisons between the two groups. *MADRS*, Montgomery-Äsberg Depression Scale; *N*, number.

**Figure 3** depicts the absolute changes in BAI, SDS, and MADRS total scores at 6-month follow-up with comparisons between the two groups. The absolute changes were comparable between the two groups (all *P*>0.05). There was a trend towards a higher absolute change in SDS total score in the control group (*P*=0.064). Specifically, the average 6-month follow-up BAI total score decreased by -42.9% in the intervention group and by -30.4% in the control group. Regarding the SDS scale, the intervention group improved by -25.4% and the control group by -27.1%. The total score of MADRS decreased by -59.4% in the intervention group, compared to a -55% decrease in the control group.

**Figure 4:**
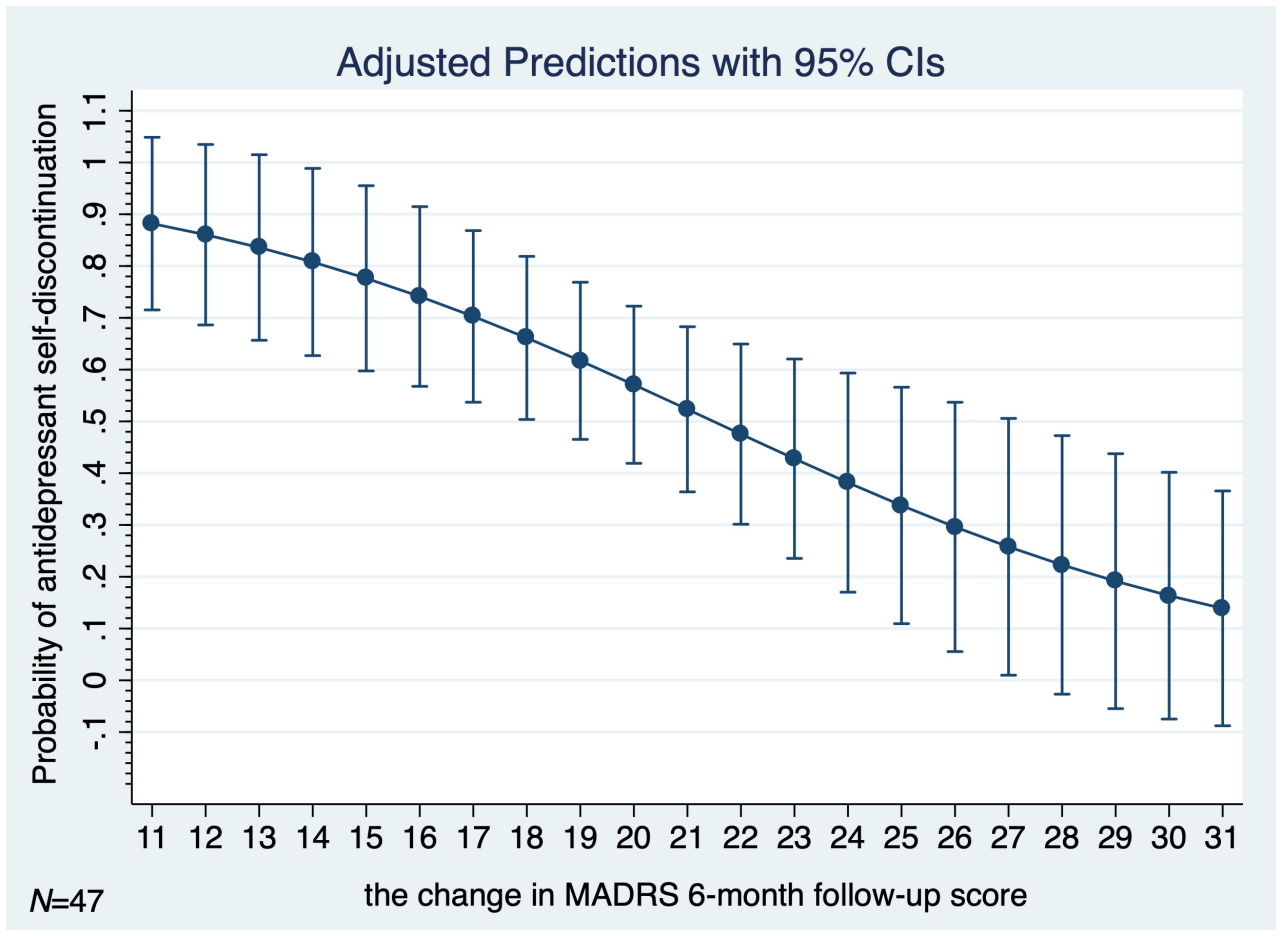
Adjusted predictions of change in MADRS total score at 6-month follow-up for probability of antidepressants self-discontinuation at 1-year follow-up in the non-educated control group (*N*=47). *MADRS*, Montgomery-Äsberg Depression Scale; *N*, number; *95% CI*, 95% confidence interval.

**Figure 4** shows the adjusted predictions of changes in MADRS total score at 6-month follow-up for probability of antidepressants self-discontinuation at 1-year follow-up in the non-educated control group (*N*=47). The graph displays the computed predicted probability of the treatment self-discontinuation with its 95% CI for the absolute change in MADRS total score at 6-month follow-up in the control group ranging from 11 to 31 points. The findings suggest that each 2–4 points of improvement in MADRS total score at 6-month follow-up are associated with an approximately 10% decrease in the probability of antidepressants self-discontinuation at 1-year follow-up in the non-educated control group.

**Figure 5:**
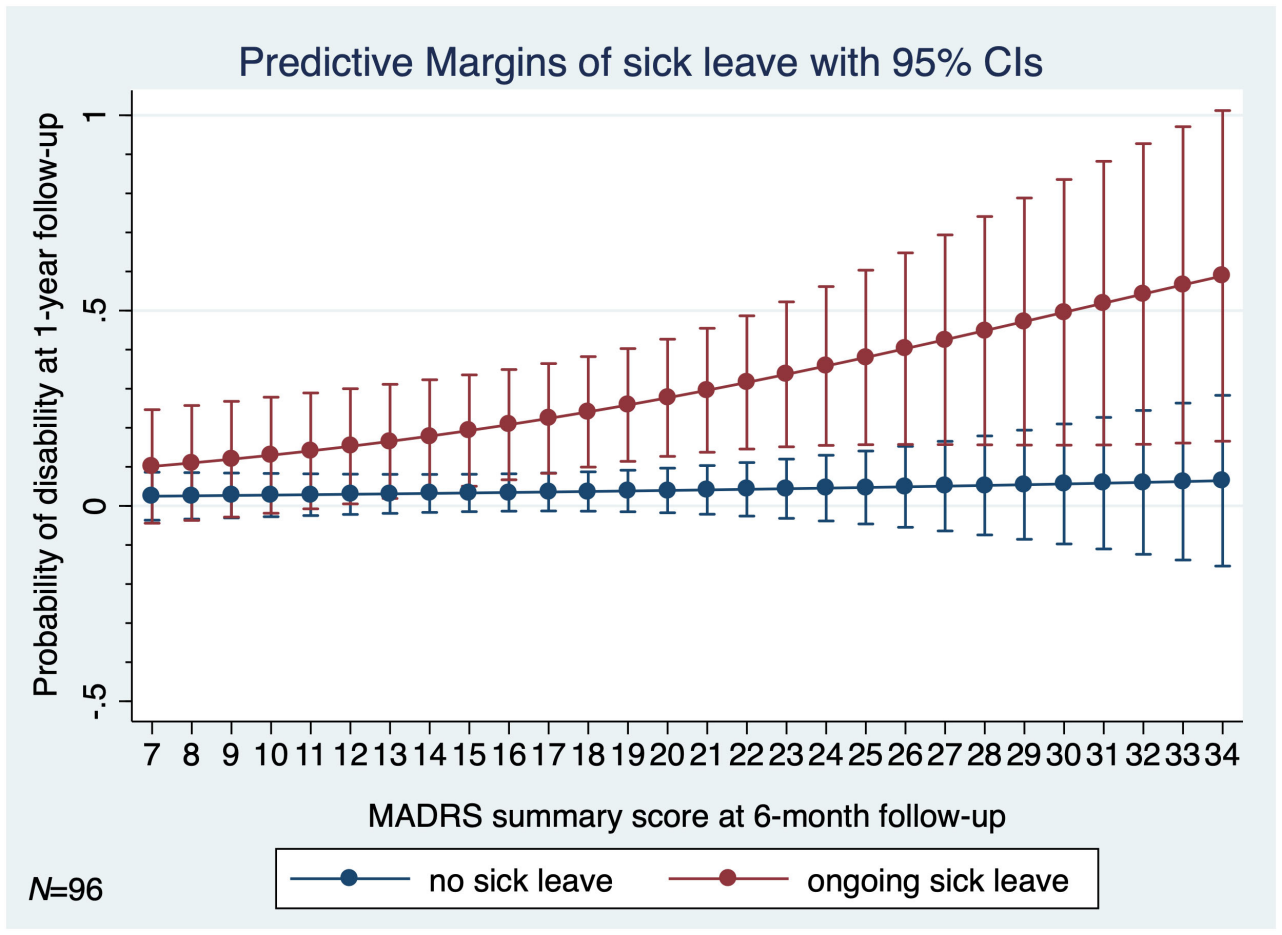
Estimated predictive margins for sick leave at 6-month follow-up for probability of disability due to MDD at 1-year follow-up (*N*=96). *MADRS*, Montgomery-Äsberg Depression Scale; *MDD*, major depressive disorder; *N*, number; *95% CI*, 95% confidence interval.

There was a considerably higher rate of the sick leave among participants in the non-educated control group (*P*<0.001). The disability due to MDD at 1-year follow-up was predicted by the absence of the psychoeducational intervention (*P*=0.002) and by the MADRS total score at 6-month follow-up (OR 1.10; 95% CI 1.003–1.195; *P*=0.044; *N*=96). **Figure 5** graphs the estimated predictive margins for sick leave at levels of the 6-month follow-up MADRS total score from 7 through 34 for probability of disability due to MDD at 1-year follow-up. In a multivariate logistic regression model adjusting for age, sex, and marital status, both the absence of psychoeducational intervention and the ongoing sick leave predicted disability due to MDD at 1-year follow-up (*P*=0.006).

As for the qualitative results at 6-month follow-up, most participants from the intervention group stated that all intervention sessions had been very supportive, helpful, and useful for them. The subjects reported additional improvements in decision making and in organizing their daily activities. They were more successful in preventing depression, skepticism, pessimistic thoughts and perceived that “they had their life more in their hands”. They associated these improvements with skills learned at the psychoeducational sessions. Reviewing notes taken at the sessions was helpful as well. Specifically, during the third intervention session, the participants reported considerable improvements in mood and in sleep with reduction in tearfulness and envision an optimistic future. Objectively during the psychiatric examination, there were improvements in concentration and sleep with less pessimistic thoughts than before. Further, at the fourth intervention session the participants reported they were feeling less fatigued, more energized, and more inclined to help someone in need. Objectively, there were considerable improvements in ability to feel pleasure and to engage in pleasant activities.

## Discussion

The primary finding of the present study is the beneficial effect of the in-person psychoeducational intervention on clinical course of MDD in both the short and long term. Specifically, the intervention based on a modified Munoz’s Depression Prevention Course was associated with significant and clinically meaningful short-term and long-term follow-up improvements in all patient-reported outcome measures (decreases in the BAI, SDS, and MADRS total scores, lower rates of the rehospitalizations within one year and after one year, lower rates of the ongoing sickness absence, less persons with disability due to MDD at 1-year follow-up, and less subjects who self-discontinued treatment). The results confirm and substantiate findings from previous studies exploring the effects of psychoeducational programs in the treatment and prevention of depressive relapses in different settings (30–36).

Of particular interest is the dynamics of the mood improvements between the psychoeducational sessions. Specifically, between the second and the third intervention session, we have observed considerable improvements in the subscales of the MADRS questionnaires the “Concentration difficulty”, the “Reduced sleep”, and the “Pessimistic thoughts” and improvements in the subscale of the SDS item 3 “I have crying spells or feel like it” in the intervention group compared to the control group. Furthermore, between the third and the fourth intervention session, we have observed significant improvements in the subscale of the MADRS the “Inability to feel” and improvements in the subscales of the SDS item 10 “I get tired for no reason” and the item 17 “I feel that I am useful and needed” in the intervention group compared to the control group. Improvements in these symptoms are generally difficult to achieve in clinical practice. As a result, they improve more slowly and often tend to persist as residual ones. Our findings indicate that the in-person group psychoeducational intervention can be an effective tool to boost happiness and reduce anxiety.

Sex differences in psychological and physiological manifestations of MDD may contribute to the disease severity and outcomes. While MDD represents a global mental health concern, it disproportionally affects women who are more likely to be diagnosed than men. According to the American Psychiatric Association (37), women are 1.5–3 times more likely to suffer from depression. In Slovakia and in most other countries, the male-female ratio of MDD is 1:2 (38, 39). In our study, males in the intervention group had significantly greater improvements in the MADRS total score at 6-month follow-up than females. This observation was not explained by any of the examined outcomes. Significantly more subjects in the intervention group were married/in a domestic partnership and less were single or divorced compared to the control group. Given the preference of the subjects to be allocated in the intervention group, this finding may support previous observations that married individuals are more likely to volunteer, be involved in their community, and have more civic responsibility (40).

Since its inception, the psychoeducation appears to be a promising tool to improve the effectiveness of psychiatric treatments and to achieve a better prognosis even in individuals at clinical high risk for psychosis (41) and in persons with psychotic disorders (42, 43). Our findings are in good agreement with those of Pekkala and colleagues (43) who meta-analyzed 10 randomized controlled studies on psychoeducation compared to standard care to demonstrate that psychoeducation of patients with schizophrenia significantly reduced relapse or readmission rates at 9-month to 18-month follow-up. Our results also confirm previous findings that psychoeducation could have a positive impact on knowledge gain, adherence to medication, and global level of functioning (43). In our study, there was a statistically significant difference in self-discontinuation of antidepressants between the intervention group and the control group. The results are consistent with the study by van Geffen and colleagues (44), who reported 22% rate of nonacceptance of selective serotonin-reuptake inhibitor treatment. Fear of adverse effects (44) and the actual lack of education were main reasons for not accepting antidepressants.

One of the major scientific challenges of the twenty-first century is the prevention of mental disorders and their unfavorable clinical course. Managing depression is the bread and butter of psychiatry (45). The acute and the maintenance treatment of MDD consist of pharmacotherapy and psychological approaches such as psychoeducation and adherence monitoring (46). Several treatments have been developed, particularly those of a pharmacotherapeutic and psychotherapeutic nature, which have been shown to be effective in ameliorating acute episodes of MDD. What remains to be addressed is the challenge of preventing recurrence after an acute episode abates. Relapse and recurrence of depressive disorders are common in clinical psychiatry. They contribute to an increased need of medical care, sickness absence, and hospitalizations. Therefore, they require further attention. Similar to report of Young’s intervention based on interpersonal psychotherapy model (47) is our finding of considerable differences in relapses of depression leading to rehospitalizations within 1 year (2.1% vs. 16.7%; *P*<0.001) and to rehospitalizations after 1 year (6.3% vs. 25%; *P*<0.001). Particularly for the treatment of recurrent depressive disorder Keller and colleagues (48) recommend the combination of pharmacotherapy, psychotherapy, and psychoeducation.

The current evidence from controlled studies suggests that even short-lasting psychotherapeutic and psychoeducational programs are safe and effective in treating and preventing depression (30–33). The psychoeducational intervention in our study was short-lasting and completely delivered over a 1-month period. Our findings confirm previous results (34) and extend them beyond increasing participants’ knowledge to a positive effect on medication adherence. The important finding of the present study is that subjects in both groups improved the self-reported anxiety, the self-reported depressive symptoms, and the clinician-rated depressive symptoms in the short term (up to six months), while continuing the usual care with antidepressant medication. This observation can be explained by the fact that all subjects were taking antidepressants during the first six months following the baseline psychiatric hospitalization discharge, and this antidepressant treatment is effective without the necessity of ongoing psychoeducation. The findings also suggest that pharmacotherapy of MDD is safe for the short-term treatment of MDD. This is consistent with previous findings (1). On the other hand, from the long-term perspective, the psychoeducation gains importance as a tool to improve the overall functioning, adherence to treatment, and relapse prevention. The analyses of rates of rehospitalizations, disability, and treatment self-discontinuation after six months following the hospitalization discharge provided evidence of the benefit of adding psychoeducation to usual care in the medium term (6–12 months) and long term (longer than 12 months). The lower rates of sickness absence and disability in the intervention group favored the addition of psychoeducation to usual care with antidepressant medication compared with usual care alone. The differences between the two groups in rates of rehospitalizations, disability, and treatment self-discontinuation after six months indicate that the psychoeducational intervention had a positive impact on persons with MDD beyond the short-term effects. It appears that this effect is mediated by changes in participants’ thinking and activity patterns as being a result of the adoption of psychoeducational techniques and a modified lifestyle.

Globally, the present findings correlate favorably with the results of Linde and colleagues (35), who meta-analyzed 37 studies with psychological treatments in 7,024 patients. The meta-analysis has demonstrated that the face-to-face cognitive behavioral therapy (CBT; OR 1.80; 95% credible interval 1.35–2.39), face-to-face counselling and psychoeducation (1.65; 1.27–2.13), remote therapist lead CBT (1.87; 1.38–2.53), guided self-help CBT (1.68; 1.22–2.30) and no/minimal contact CBT (1.53; 1.07–2.17) were superior to usual care or placebo, but not face-to-face problem-solving therapy and face-to-face interpersonal therapy in primary care (35). Findings suggest that psychological interventions in particular those with a cognitive behavioral approach are promising. While psychoeducation is a simple and illness-focused therapy with prophylactic efficacy in all major mood disorders, it assumes a proper setting, including open-door policy, team effort and empowerment of the therapeutic alliance (31).

The psychoeducational intervention based on a modified Munoz’s Depression Prevention Course has been chosen because it is one of the most complex, most comprehensible, and easily adoptable educational programs that consists of both theoretical and practical parts. The intervention includes some elements of cognitive behavioral therapy along with some specific features. Specifically, the intervention comprises social learning, targets acquiring self-control strategies, cognitive techniques for dealing with depressogenic thoughts, and planning and developing enjoyable activities. It also supports an ongoing training of social skills, social competences, planning the future, and dealing with expected life events. To facilitate the process, the subjects also utilize activity worksheets and work on practice sheets.

In brief, the present study has demonstrated that the in-person psychoeducational intervention based on a modified Munoz’s Depression Prevention Course was effective in reducing depression levels in adults recently discharged from a psychiatric hospitalization. The psychoeducational intervention we used appear to produce clinically and statistically significant improvements of the clinical course of MDD. The subjects learned methods to gain greater control over their mood, specifically by increasing pleasant activities, social skills training, and cognitive approaches. This observation concurs well with Zeiss and colleagues (36). Only six out of 54 participants in the intervention group had not completed at least three sessions and were therefore excluded from analyses. We find this pattern of results encouraging. The results lend support to targeting the in-person approach that promotes participation, interaction and collaboration, personalized learning, and doubt resolution. Yet, the intervention is time consuming and human demanding. As reported by Dowrick and colleagues (49), the evidence we found points to effectiveness of group psychoeducation in prevention of depression in healthcare and community settings. The findings implicate that psychoeducation should be a standard part of the treatment of depression, as it significantly contributes to an improved clinical course of MDD. Based on the current observations, the modified Munoz’s Depression Prevention Course appears to be especially efficient for preventing depressive disorders. Substantial evidence suggests that depression is preventable (50, 51). Given the existing barriers, innovative depression prevention programs are needed.

One of the strengths of the present study is that it represents a comprehensive longitudinal examination of the adults with MDD recently discharged from a psychiatric hospitalization based on the in-depth analysis of the selected demographic and clinical variables controlling for key covariates in analyses. Selection of the follow-up outpatient data avoids bias that may potentially result from the sole use of inpatient data and improves the generalizability of the results to the target population. Another strength of the study is the detailed processing and dedication to patients by professionals. A certain disadvantage is that the study was not randomized. This is mainly because randomization between preferring a treatment/intervention and not is impossible. Therefore, the group allocation was based on the preference of each subject. Importantly, the absence of randomization did not produce groups that would differ in important ways. We did not observe any imbalance in baseline prognostic factors between arms of this non-randomized study. Another limitation was the relatively small sample size. To avoid the bias and improve the reliability of results, only subjects with stable symptoms of nonpsychotic depression without any psychiatric or somatic comorbidity hampering the comprehension and following of the study were included. Of note, subjects with psychotic symptoms and/or with a higher risk of suicide were excluded given the policy and ethical considerations. Individual data about smoking, physical activity, or other direct lifestyle measurements were unavailable. Also, detailed information about the types of antidepressants was not considered in analyses. The study did not report the number of persons with different types of antidepressants that could enable to further investigate differences in outcomes. The findings should be interpreted with caution also because of possible confounding by other unmeasured factors. Yet, it was a pragmatic study, and all subjects had a usual care with pharmacological treatment of MDD. Specific underlying mechanisms of depressive relapse and depressive recurrence remain not completely understood and warrant additional studies using longitudinal sociodemographic and clinical data. Further research is needed to demonstrate the effectiveness of the depression prevention program delivered in a range of settings including different ethnic and cultural groups by a range of practitioners.

## Data availability statement

The raw data supporting the conclusions of this article will be made available by the authors, without undue reservation.

## Ethics statement

The studies involving humans were approved by the independent ethics committee of the Presov self-governing region. The studies were conducted in accordance with the local legislation and institutional requirements. The participants provided their written informed consent to participate in this study.

## Author contributions

DB: Conceptualization, Investigation, Methodology, Project administration, Resources, Supervision, Writing – original draft, Writing – review & editing. MP: Conceptualization, Formal analysis, Investigation, Methodology, Software, Writing – original draft, Writing – review & editing. LI: Investigation, Writing – review & editing. MK: Investigation, Writing – review & editing.
